# Feasibility of a randomised trial of a continuing medical education program in shared decision-making on the use of antibiotics for acute respiratory infections in primary care: the DECISION+ pilot trial

**DOI:** 10.1186/1748-5908-6-5

**Published:** 2011-01-18

**Authors:** Annie LeBlanc, France Légaré, Michel Labrecque, Gaston Godin, Robert Thivierge, Claudine Laurier, Luc Côté, Annette M O'Connor, Michel Rousseau

**Affiliations:** 1 Knowledge Transfer and Evaluation of Health Technologies and Interventions Unit, Research Centre of the Centre Hospitalier Universitaire de Québec, Québec, Canada; 2 Department of Family and Emergency Medicine, Université Laval, Québec, Canada; 3 Faculty of Nursing, Université Laval, Québec, Canada; 4 Faculty of Medicine, Université de Montréal, Québec, Canada; 5 Faculty of Pharmacy, Université de Montréal, Québec, Canada; 6 Faculty of Health Sciences, School of Nursing, University of Ottawa, Ottawa, Canada

## Abstract

**Background:**

The misuse and limited effectiveness of antibiotics for acute respiratory infections (ARIs) are well documented, and current approaches targeting physicians or patients to improve appropriate use have had limited effect. Shared decision-making could be a promising strategy to improve appropriate antibiotic use for ARIs, but very little is known about its implementation processes and outcomes in clinical settings. In this matter, pilot studies have played a key role in health science research over the past years in providing information for the planning, justification, and/or refinement of larger studies. The objective of our study was to assess the feasibility and acceptability of the study design, procedures, and intervention of the DECISION+ program, a continuing medical education program in shared decision-making among family physicians and their patients on the optimal use of antibiotics for treating ARIs in primary care.

**Methods:**

A pilot clustered randomised trial was conducted. Family medicine groups (FMGs) were randomly assigned, to either the DECISION+ program, which included three 3-hour workshops over a four- to six-month period, or a control group that had a delayed exposure to the program.

**Results:**

Among 21 FMGs contacted, 5 (24%) agreed to participate in the pilot study. A total of 39 family physicians (18 in the two experimental and 21 in the three control FMGs) and their 544 patients consulting for an ARI were recruited. The proportion of recruited family physicians who participated in all three workshops was 46% (50% for the experimental group and 43% for the control group), and the overall mean level of satisfaction regarding the workshops was 94%.

**Conclusions:**

This trial, while aiming to demonstrate the feasibility and acceptability of conducting a larger study, has identified important opportunities for improving the design of a definitive trial. This pilot trial is informative for researchers and clinicians interested in designing and/or conducting studies with FMGs regarding training of physicians in shared decision-making.

**Trial Registration:**

Clinicaltrials.Gov NCT00354315

## Background

The misuse and limited effectiveness of antibiotics for acute respiratory infections (ARIs) are well documented, and current approaches targeting physicians or patients to improve appropriate use have had limited effect [1-4]. Only a few interventions combining physician, patient, and public education have been successful in reducing antibiotic prescribing for inappropriate indications [2]. In this regard, shared decision-making (SDM) could be a promising strategy to improve appropriate antibiotic use for ARIs [5].

SDM is a process by which a healthcare choice is made by physician together with the patient and is characterised by a two-way exchange of information, values, and preferences, both parties taking steps to build a consensus and reach an agreement on the decision to be made [6,7]. From a patient's perspective, SDM interventions or programs (*i.e*., patient decision aids) have been found to improve knowledge of the options and accuracy of the perception of their benefits and harms, to reduce difficulty with decision-making, and to increase participation in the process [8]. Although expanding very rapidly, SDM has not yet been adopted by physicians, and very little is known about its implementation processes and outcomes in clinical practices [9,10].

Decision-making occurring during a clinical encounter between a patient and a physician is a complex and highly interactive process. Although many studies have attempted to capture the relational nature of this encounter, members of this dyad have been mostly studied independently, as if living in different worlds [8,10,11]. Recent findings, however, have shown that combining patients' and their physicians' perspectives could enlighten the relational process occurring in the encounter [12-14]. These findings suggest the need for a better understanding of the impact of approaches such as SDM, in which the involvement of both members of the physician-patient dyad is emphasized. To our knowledge, only a few studies have reported on the difficulties pertaining to the implementation of this type of approach [15-17]. One of the critical issues in this regard consists of recruiting participants [15]. Indeed, recruiting and retaining physicians as study participants have been shown to be difficult, and even more so when it also involves recruiting their patients [15-17]. Moreover, simultaneous data collection at the point of care in physician-patient dyads generates further challenges, such as having physicians complete questionnaires regarding each recruited patient.

Pilot studies, in providing critical information on both the processes and outcomes, offer a unique opportunity to identify the difficulties of evaluating an intervention, and thus enhance the rigour of larger full-scale studies. There is growing interest in the use of pilot studies for randomised trials [18-20], particularly if the planned intervention is offered in pragmatic settings, that is, when trials are designed to inform decisions about practice [21] or when multifaceted interventions are at play [22-24]. Indeed, Campbell and colleagues (2000) [22] have recommended a stepped approach to the development and evaluation of complex interventions, thus encouraging exploratory and developmental research projects. Similarly, the US Department of Veterans Affairs Quality Enhancement Research Initiative has integrated pilot studies into their implementation process [23]. Many granting agencies now require pilot trials as safeguards to ensure that future trials are designed optimally and can be implemented in practice [25,26]. However, although the importance and necessity of pilot studies have been reported by many authors and institutions, there is a lack of frameworks or recommendations on how to conduct, assess, and interpret these studies [27-29].

In general, a *pilot study *is defined as a small-scale version of anticipated research [18,19]. More specifically, *pilot work *has been referred to as any background exploratory research that may form the basis of a future study [28,29], whereas the terms *pilot studies *or *trials *have been recommended for studies with a specific hypothesis, objective, and methodology, the latter including a randomisation procedure [28]. Feeley and colleagues (2009) have recently proposed that the objectives of pilot trials should relate to the feasibility and acceptability of the intervention, study design and procedures, and the determination of effect sizes [20]. According to these authors, feasibility is primarily concerned with the researcher's ability to execute the plan (delivery), that is, to provide the intervention and complete the study procedures, whereas acceptability concerns the suitability of the intervention (uptake) or the research design from the perspective of the participants [20]. Whether there are many or few objectives in a pilot trial, the importance is that specific objectives be established along with threshold criteria for claiming success [18].

The current paper describes the recruitment process and reports on the feasibility and acceptability of conducting a larger clustered randomised trial of the DECISION+ program, an innovative, theory-driven continuing medical education (CME) program in SDM on the optimal use of antibiotics for ARIs in primary care settings among family physicians and their patients [30]. Specifically, the objectives of this pilot trial were to assess (a) recruitment of family medicine groups (FMGs), family physicians, and patients in the study; (b) participation of family physicians to the DECISION+ program; and (c) satisfaction of participants regarding the DECISION+ program. The development, adaptation, and validation of the DECISION+ program and related material and its potential impact are reported elsewhere [30,31].

## Methods

### Study design

**Figure 1:**
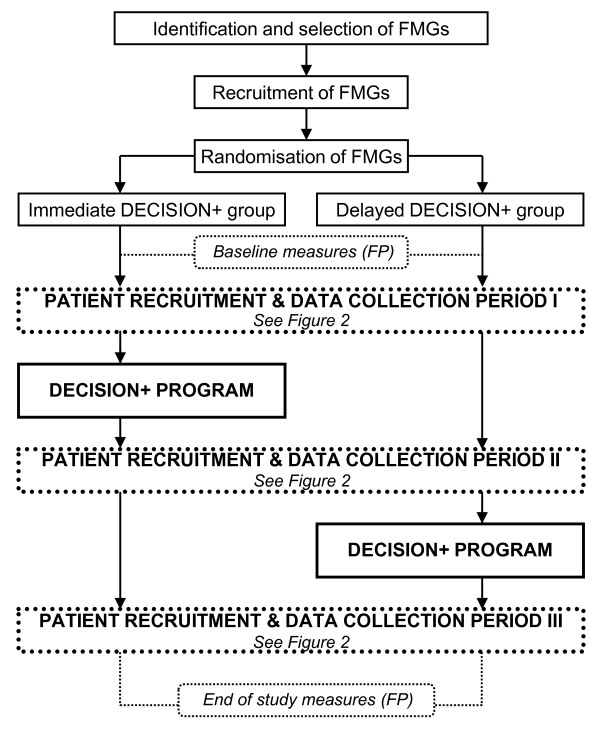
**Study design**. FMG = family practice group; FP = family physician.

This study was a two-arm pilot clustered randomised trial. FMGs, the unit of randomisation, were simultaneously and randomly assigned using an Internet-based software to either an experimental group in which participating family physicians were provided the DECISION+ program immediately or a control group in which exposure to the DECISION+ program was delayed (Figure 1). The DECISION+ program is a multifaceted intervention offered over a four- to six-month period that includes three 3-hour interactive workshops, reminders of the expected behaviours, and feedback.

### Setting

The study was conducted in FMGs of Quebec City and surrounding areas (Québec, Canada) between January 2007 and March 2008. FMGs are groups of family physicians (here referring to both family physicians and general practitioners) who work in close cooperation with nurses to offer family medicine services to registered individuals. During the last decade, in the province of Québec, the Ministry of Health and Social Services has provided incentives and support to private practitioners to construct such primary care groups in order to improve accessibility and continuity of care and facilitate interdisciplinary collaborations [32].

### Study participants

FMGs were eligible if located in two pre identified health regions (Capitale Nationale and Chaudière-Appalaches) and if their family physicians were being paid fee-for-services (in order to obtain data on medical service use from the provincial claims data bank). Family physicians from participating FMGs were excluded if they were currently involved or had previously participated in an implementation study of SDM in clinical practice, or if they did not plan to practice for the duration of the study (*e.g*., pregnancy, retirement, practice restricted exclusively to administrative duties). Patients, adults or children, were included in the study if consulting a participating family physician for an ARI and if an antibiotic treatment was being considered by either the patient (or guardian) or the family physician. Patients with a condition requiring emergency care were excluded. Patients could take part only once in the study. All participants, family physicians and patients, signed an informed consent form approved by the ethics committee from the university hospital institutional review board.

### Study procedures

#### Identification and selection of FMGs, family physicians, and their patients

FMGs are accredited by the Ministry of Health and Social Services of the Province of Québec. A list of all accredited FMGs is available, free of charge, from the Ministry's website and is updated periodically [33]. FMGs from the two selected health regions were retrieved and constituted the sample base for this project.

#### Recruitment strategies for FMGs, family physicians, and their patients

The medical director of each FMG was contacted in random order by one of the two principal investigators (FL and ML) after an initial introduction phone call by a research assistant. When contacted, the director was provided with information regarding the DECISION+ program, and a one-page summary of the project was faxed. The director was requested to discuss potential participation in the project with his/her colleagues. An FMG could either accept or decline to participate at this point. However, a short meeting between the research team and FMG members was offered up-front to all directors in order to facilitate decisions about participation in the study. All members of the FMG, including nurses and practice managers, were invited to participate in this meeting. One of the principal investigators and one or two research team members subsequently attended the meeting to present the study to family physicians and to clarify concerns or ambiguities regarding the program or the project. If at this point an FMG confirmed its participation, attending family physicians immediately completed the consent form. Additional information and consent forms were provided for family physicians not attending the meeting, and they were personally contacted by a research assistant one and two weeks later if they had not returned the consent form. Those who had still not responded after two weeks were contacted personally by one of the two principal investigators. If a given FMG decided to delay its acceptance or refusal to participate in the study, the research assistant or one of the principal investigators would contact the medical director once a week until receiving a final answer. Participating family physicians were offered CME credits upon the completion of the program and the opportunity to keep all the materials and tools offered during the project. No financial incentive was offered during the study beyond the usual Provincial Government subsidy provided to all family physicians participating in a three-hour-a-day-accredited CME activity.

Fifteen patients per family physician were recruited, five per data collection period (Figure 1). Family physicians were not directly involved in the recruitment of patients. They were recruited during walk-in clinic hours at each participating FMG by research assistants that were stationed in the FMG waiting room. Posters were displayed in the waiting room and flyers were available at the registration office to facilitate the recruitment process. Patients interested in the trial met with the research assistant in order to verify eligibility for the study. Patients had to be seeking a consultation for an ARI (acute otitis media, acute bronchitis, acute sore throat, or acute rhinosinusitis) and considering the use of antibiotics in order to be eligible for the study. No incentives or privileges were offered to participating patients.

#### Retention strategies and burden to participants

The disruption of office routine was kept to a minimum. Research assistants were responsible for all stages of the recruitment process and served as the primary means of communication between the research team and the FMG manager and participating family physicians. They maintained regular contact (*i.e*., phone calls, emails, personal visits) with the practice manager, the medical director of the FMG, and reception staff to sustain and reinforce commitment to the project. The research team provided a biweekly bulletin to keep FMGs and their family physicians informed about the progression of the study and about any recent findings regarding antibiotic use for ARIs, when available. The research team ensured that the questionnaires, when possible, were completed during a planned activity (*e.g*., workshops/staff meeting).

#### Intervention (the DECISION+ program)

The intervention was based on a conceptual framework integrating the principles of SDM, including evidence-based medicine and patients' involvement in decisions [30]. According to this framework, greater understanding of the probabilistic nature of bacterial infection in ARIs, better knowledge and communication of the benefits and risks, and active participation of the patient in the decision-making process should lead to SDM. In turn, this should favor optimal prescription of antibiotics by physicians and uses of antibiotics by patients and, therefore contributing to better health outcomes.

The development of the program was under the supervision of the principal investigators, two family physicians (an epidemiologist expert in evidence-based medicine and an expert in SDM and its implementation in primary care), in collaboration with two CME university offices. All three workshops and material were pretested with a panel of family physicians and experts from various related fields (*e.g*., anthropology, medical education, epidemiology, education, and medicine).

#### Workshops

A series of three 3-hour workshops were offered over a period of four to six months to participating family physicians. The workshops were based on learning principles in medical education and addressed specific key components of the study framework [30]. They were adapted from previous work by one of the principal investigator [13]. The first workshop focused on the probabilistic nature of the diagnosis of a bacterial versus viral ARI. The second workshop focused on the available evidence regarding risks and benefits when facing a decision about whether to use antibiotics in ARI. It also addressed effective strategies to communicate this information to patients. The third workshop focused on strategies to foster active participation of patients in the decision-making process. Each workshop included videos and reflective exercises that facilitated group discussion. Various decision-making support tools as well as a toolkit that contained the materials of the workshop were also made available to the participants. The workshops were led by the two principal investigators, accompanied by a research assistant.

#### The decision support tools

The decision support tools included four sections: (1) a diagnostic aid for physicians to better appraise the probabilistic nature of the diagnosis of a bacterial versus viral ARI, (2) a graphic display of the benefits and risks of antibiotic use for ARIs to be shared with their patients, (3) a section helping patients to elucidate their values and preferences regarding the benefits and risks of using or not using antibiotics, and (4) a section regarding the decision to be made with the patient. The first two sections were based on the best scientific evidence regarding the probabilistic nature of the diagnosis of an ARI and the effectiveness of antibiotics for treating ARIs. The remaining sections were adapted from the O'Connor generic decision aid [34] and included the SURE tool, which assesses the comfort with the decision that was made [35]. Family physicians were given the first section of the decision support tools at the first workshop and were strongly encouraged to try it out with their patients before the next workshop. The other sections were subsequently added during the subsequent workshops with the same recommendations. A global decision support tool that integrated all sections was provided at the end of the program.

#### Reminders of expected behaviours and feedbacks

**Figure 2:**
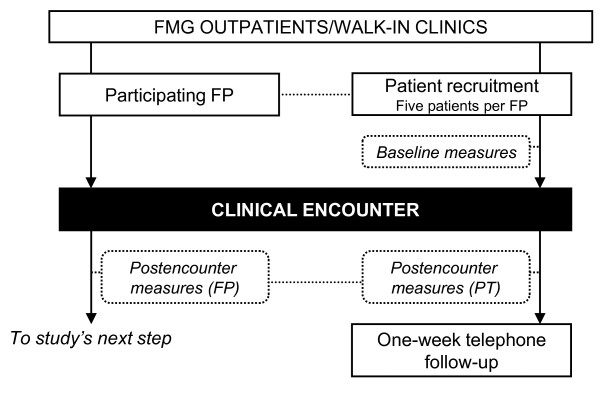
**Patient recruitment and data collection periods I, II, & III**. FMG = family practice group; FP = family physician; PT = patients.

Aside from the biweekly progression bulletin, one-sheet letter-size paper reminders reemphasizing the use of the decision support tools discussed in a previous workshop were sent to participating family physicians every one to two weeks after the first and second workshops. During the third workshop, family physicians were provided with individual feedback on the agreement with their patients' evaluations on comfort with the decision to use or not to use antibiotics in ARI. In addition, they were informed of the pooled results from their colleagues. Time was made available during the workshop to explain how to interpret the results. This information was collected at the time of the first data collection period (the first five patients per physician) prior to exposure to the workshop (Figure 2).

#### Participation and satisfaction of family physicians regarding the intervention

To facilitate attendance, workshops were offered at the site of each participating FMG. Family physicians not available at that time were given the opportunity to attend the workshop of another FMG within their study group (immediate or delayed). All family physicians received copies of the program material. Family physicians were considered to be collaborators and were invited to give their insights. This resulted in open discussions regarding logistical aspects of the project following each workshop. Family physicians also completed a questionnaire regarding their level of satisfaction with the CME program, and they had the opportunity to express anonymous comments on the project.

#### Data collection

Data about the organisation were collected from participating FMGs at the beginning of the study. Modifications to the FMG status or staff or significant events occurring in the participating FMG throughout the study period were also recorded. Sociodemographic variables along with entry/exit measures were collected from family physicians through self-reporting questionnaires before the first and after the last patient recruitment period (Figure 1). Postclinical encounter data were collected from family physicians for each of their recruited patients during each recruitment period (Figure 2). Questionnaires were attached to patient's medical records, which encouraged family physicians to complete them at the same time. Patient data were collected using self-reporting questionnaires completed before and after the consultation and one week after the consultation by telephone interview (Figure 2).

#### Outcome measures

Feasibility (delivery) and acceptability (uptake) of the DECISION+ program were the main outcome measures of this pilot trial. Investigators had established *a priori *threshold for specific feasibility and acceptability criteria. These were the following: (a) the proportion of contacted FMGs participating in the pilot study would be 50% or greater, (b) the proportion of recruited family physicians participating in all three workshops would be 70% or greater, (c) the mean level of satisfaction from family physicians regarding the workshops would be 65% or greater, and (d) the proportion of missing data in each completed questionnaire would be less than 10%.

Satisfaction with the program was measured using a standardised CME satisfaction questionnaire from the CME university office. This questionnaire contains 13 items, each assessed on a four-point (1 = completely disagree to 4 = completely agree) Likert-scale, related to the relevance and scientific quality of the content, the educational structure of the workshops, the trainers, and the workshops in general.

After the onset of the trial, investigators and the research team met regularly (biweekly or monthly steering group meetings) to evaluate the progress of the study and to overcome or discuss issues and barriers that had arisen. These evaluations were based on reports from on-site research assistants and comments and evaluation from trainers and participants in the workshops. Thus, retention and burden outcomes were added to the preestablished outcomes. The appropriateness of selection, timing, and sequencing of similar study measures for patients and their family physicians were also documented.

Secondary outcome measures are detailed and reported elsewhere [30,31]. Briefly, both family physicians and patients completed similar questionnaires pertaining to the decision to use or not to use antibiotics, their comfort with the decision (Decisional Conflict Scale) [36], and their intention to engage in SDM in future encounters for ARIs [30]. Family physicians further reported their intention to use clinical practice guidelines pertaining to the use of antibiotics in ARI. Patients reported on their regrets regarding the decision that was made (Decisional Regret Scale) [37]. The prescription profile of antibiotics in ARIs of participating family physicians was also collected from the provincial registry for patients covered by the public drug plan.

## Results

### Identification, selection, and recruitment of participants (feasibility)

**Figure 3:**
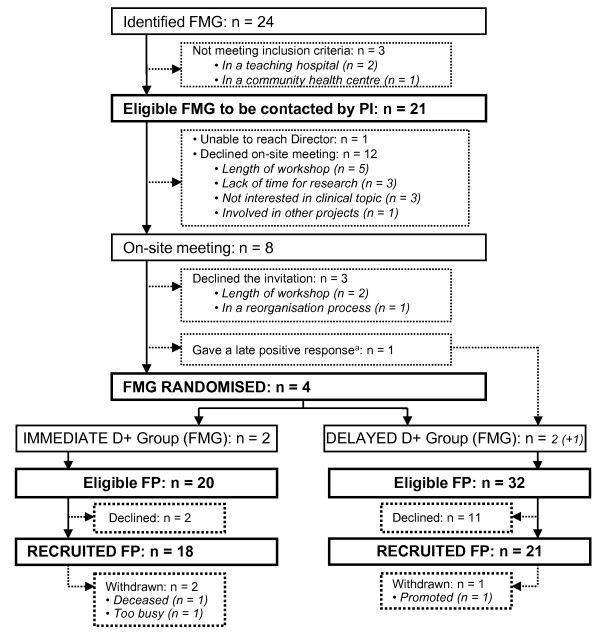
**Recruitment and retention of FMGs and family physicians**. ^a^This FMG was not randomised in order to avoid breaking the allocation of concealment. FMG = family medicine group; D+ = DECISION+ pilot trial; FP = family physician; PT = patients; PI = principal investigator.

According to the list extracted from the Ministry of Health and Welfare web-based registry, there were 24 accredited FMGs in the two selected health regions at the time of recruitment. In total, 21 FMGs were eligible (Figure 3).

A research assistant made 41 introductory phone calls to contact the medical directors of the 21 eligible FMGs over a four-week period. One director could not be contacted. Information leaflets were faxed to the 20 contacted FMGs. Second attempts by the research assistant to assess willingness to participate in the study or to attend an information meeting resulted in 39 calls over an 11-week period. Twelve FMGs declined participation in the study and eight FMGs agreed to meet with the research team. After the meeting, a total of 36 additional calls over a period of nine weeks were necessary to get a final answer. Four FMGs agreed to participate. Two were randomly assigned to the experimental group (immediate DECISION+ program) and two to the control group (delayed DECISION+ program). A fifth FMG accepted too late to be randomised and was assigned to the control group, for a total of five (24%) contacted FMGs participating in the study (Figure 3).

Out of the 52 eligible family physicians working in the five participating FMGs, 39 (75%) agreed to participate in the study. Proportions of participating family physicians in the two experimental and three control FMGs were 90% and 66%, respectively. The characteristics of family physicians by study groups are detailed in Table 1.

**Table 1:** Characteristics of participating family physicians (n = 39)

Characteristics	Immediate DECISION+ (n = 18)	Delayed DECISION+ (n = 21)	Total
Age, yrs (mean ± SD)	48 ± 9	49 ± 7	48 ± 8
Years in practice (mean ± SD)	22 ± 9	23 ± 9	22 ± 9
Hours/week of professional activities (n = 36) (mean ± SD)	45 ± 11	46 ± 13	46 ± 12
Number of patients per week (mean ± SD)	105 ± 47	113 ± 29	109 ± 38
			
Women (%)	10 (56)	10 (48)	20 (51)
			
Preference of decision-making style [45]			
Patient alone	4 (22)	0 (0)	4 (10)
Patient after considering physician's opinion	4 (22)	9 (43)	13 (33)
Patient and physician	3 (17)	4 (19)	7(18)
Physician after considering patient's opinion	6 (33)	8 (38)	14(36)
Physician alone	1 (6)	0 (0)	1(3)

SD = standard deviation.

A total of 544 patients were recruited, and 510 (94%) were contacted one week after the medical visit. On average, there were 5.3 patients recruited per family physician per period per group. Patients' characteristics are reported in Table 2. The first patient recruitment period lasted 9 weeks and took place during the winter of 2007, while the second and third periods lasted, respectively, 17 and 9 weeks and took place during the summer of 2007 and winter of 2008.

**Table 2:** Patients' characteristics

	Patient recruitment phases		
Characteristics	Phase I	Phase II	Phase III
Total number of patients	199	181	164
Adults/Children ratio (<14 years old)	136/63	118/63	124/40
			
Age, years (mean *± *SD)			
Children (<14 years old)	5 ± 4	6 ± 4	4 ± 4
Adults	42 ± 14	39 ± 15	40 ± 13
			
Male, frequency (%)			
Children (<14 years old)	33 (52)	36 (57)	21 (53)
Adults	42 (31)	42 (36)	36 (29)
			
Health condition			
Very good	71 (35.7)	94 (52.0)	73 (44.5)
Average	96 (48.2)	69 (38.0)	71 (43.3)
Problematic	31 (15.6)	18 (10.0)	20 (12.2)
Did not respond	1 (0.5)		
			
Education level			
High school or less	87 (43.7)	70 (38.7)	58 (35.4)
College	62 (31.2)	54 (29.8)	55 (33.5)
University	49 (24.6)	54 (29.8)	49 (29.9)
No response	1 (0.5)	3 (1.7)	2 (1.2)
			
Employment status			
Working	147(73.9)	139 (76.8)	137 (83.5)
Not working	17 (8.5)	7 (3.9)	2 (1.2)
Other	17 (8.5)	9 (4.9)	10 (6.1)
No response	18 (9.1)	26 (14.4)	15 (9.2)
Family income			
<$15,000	16 (8.0)	23 (12.7)	6 (3.7)
$15,000-$29,999	34 (17.1)	17 (9.4)	18 (11.0)
$30,000-$44,999	37 (18.6)	31 (17.1)	25 (15.2)
$45,000-$59,999	25 (12.6)	31 (17.1)	27 (16.5)
≥$60,000	79 (39.7)	70 (38.7)	76 (46.3)
No response	8 (4.0)	9 (5.0)	12 (7.3)
Type of health insurance, private^a^	149 (74.9)	118 (65.2)	131 (79.9)
Did not respond			1 (0.6)
Preferred role in decision making			
Patient alone	9 (4.5)	10 (5.5)	7 (4.3)
Patient after considering physician's opinion	62 (31.2)	71 (39.2)	54 (32.9)
Patient and physician	55 (27.6)	39 (21.6)	37 (22.6)
Physician after considering patient's opinion	47 (23.6)	39 (21.6)	38 (23.2)
Physician alone	22 (11.1)	22 (12.1)	26 (15.8)
Did not respond	4 (2.0)		2 (1.2)

^a^All others are public health insurance.

### Retention of participants in the study and data collection (feasibility and acceptability)

All five FMGs completed the study. A total of 36 (92%) family physicians completed the study (Figure 3). All family physicians and patients completed the baseline questionnaire and the questionnaires after each clinical encounter. Thirty (83%) of the 36 family physicians that completed the study completed their end-of-study questionnaire. The proportion of missing items in the completed questionnaires was less than 8% for each data collection period.

### Intervention (feasibility)

A total of 15 workshops were offered, for a total of 45 hours. Five of the six workshops in the experimental group (immediate) were offered by both principal investigators, whereas the remainder was offered by one of the principal investigator and a collaborator in the study. None of the workshops was reassigned. In the control group (delayed), only one of the nine workshops was offered by both principal investigators. Six workshops were offered by one principal investigator and a collaborator, one was offered by one principal investigator only, and one was conducted by two collaborators. Two workshops had to be reassigned because of anticipated poor attendance or the unavailability of principal investigators or collaborators.

### Participation and satisfaction of family physicians (acceptability)

Overall, 29 (74%) family physicians participated in the first workshop, 27 (69%) in the second workshop, and 22 (56%) in the third workshop (Table 3). Although 27 (69%) family physicians attended two or more workshops, the proportion of recruited family physicians who participated in all three workshops was 46%. Details of participation according to study groups are presented in Table 3.

**Table 3:** Attendance and numbers of workshops completed by family physicians

	Attendance at the workshops n (%)			Number of workshops completed n (%)			
	Workshop 1	Workshop 2	Workshop 3	0	1	2	3
**Immediate D+ group (n = 18)**	15 (83)	13 (72)	11 (61)	1 (6)	4 (22)	4 (22)	9 (50)
**Delayed D+ group (n = 21)**	14 (67)	14 (67)	11 (52)	5 (24)	2 (9)	5 (24)	9 (43)
**Total**	29 (74)	27 (69)	22 (56)	6 (16)	6 (16)	9 (23)	18 (46)

D+ = DECISION+ continuing medical education program.

Global mean levels of satisfaction for the workshops in the immediate and delayed groups were, respectively, 97% and 98% for the first workshop, 96% and 90% for the second workshop, and 95% and 89% for the third workshop. The overall level of satisfaction of family physicians for the workshops of the DECISION+ program was 94%. The results according to the various items evaluated were similar to global mean levels and, thus, are not reported in detail.

### Discussion

To the best of our knowledge, this paper is among the first to describe in detail the recruitment process of FMGs, family physicians, and patients to a pilot trial of the DECISION+ program, a CME program in shared decision-making on the optimal use of antibiotics for treating ARIs in primary care practice. Moreover, the results of this trial support the feasibility and acceptability of conducting a large clustered randomised trial involving dyads of family physicians and their patients in SDM regarding the optimal use of antibiotics for ARI. This conclusion is reached even if not all predetermined standards for our criteria were always fully met. Indeed, it has been established that not reaching the preestablished criteria does not necessarily indicate unfeasibility of the trial but rather underlines changes to be made to the protocol [28].

Identification of FMGs and associated family physicians was a relatively simple process. The necessity for FMGs to be accredited, the relative stability of these FMGs, and the availability of a free web-based registry facilitated this process. Identification of individual family physicians would have been more difficult because available registries are rapidly outdated [15].

The main difficulty in recruitment appeared during the initial contact by the principal investigator with the medical director of the FMG. Twelve FMGs (57%) declined the invitation even before the face-to-face meeting. Thus, 24% of the eligible FMGs agreed to take part in the study, less than the 50% expected. We were probably too confident when targeting a 50% positive response rate from all identified FMGs. Recent studies regarding appropriate use of antibiotics reported recruitment rates for groups of providers of 56% and 37% [38,39]. Recruitment rates were poorly reported, when reported at all, in other similar studies [10,40], despite their importance in providing important information regarding generalisability of results. The main reason for refusal from the FMG was that participation would add unnecessary workload (nine hours of workshops) to already too busy family physicians or that the clinical topic was felt to be irrelevant for their CME activities at that time. In addition, the project was carried out at the time that some FMGs were in the implementation phase, and this reorganisation process might have contributed to the smaller than anticipated number of acceptance.

Nonetheless, it is interesting to note that out of the eight FMGs that agreed to an on-site meeting, four immediately accepted the invitation to participate in the study. This suggests that having the opportunity to clarify face-to-face the nature of the study and the time and efforts required from participants may facilitate the recruitment process when participants are interested in learning about the study. According to Dormandy and colleagues (2008), factors that appeared important in retaining practices in a study are good communication, easy collection methods, and payment of incentives as scheduled [41]. At the time of the first contact, the issue of incentive was not discussed with the FMG. This did not seem to affect their agreement to participate or not after the meeting, as none of the FMGs reported the lack of payment as a reason for refusal. This reinforced earlier findings showing that financial incentive alone is not sufficient for a high recruitment rate [15,17]. Moreover, the acceptance rate from family physicians from these FMGs was high. Indeed, 75% of family physicians agreed to take part in the study. It should, however, be noticed that acceptance by the FMG was probably dependant on acceptance by a large proportion of its family physicians.

Conducting studies with family physicians and their patients is challenging, mostly as a result of the competing time demands of family physicians for other clinical and administrative tasks [15,17]. Furthermore, it is one thing to take part in a study and facilitate recruitment of patients for the research team, but to have to complete documentation for each patient after each of their encounters is a major challenge. To cope with these issues, the strategies used by this research were to minimise the burden on the family physicians by having research members take care of all study-related tasks, as done in previous work [13]. This appears to have been very successful since the retention rate of all participants was very high. The number of patients per physician was also kept to a minimum.

As for patients, the challenge was to proceed to recruitment in the FMG waiting room, without disrupting or delaying the patients' consultation flow. Having research assistants recruiting patients minimised a potential selection bias if recruitment had been driven by family physicians and maximised the recruitment rate, as recruitment driven by physicians is usually only moderately successful [17].

Considering their busy schedules, we anticipated that collecting information from physicians after each clinical encounter would be challenging, and therefore, we were particularly interested in the proportion of missing postencounter questionnaires. Surprisingly, all completed the questionnaires (100% completion rate), and there were very few missing data, suggesting that it is possible to collect similar and simultaneous measures from both family physicians and their patients. Factors that might have contributed to this high rate could include the fact that postencounter questionnaires were very short (approximately two to three minutes to complete) and were included with the patient's medical file so that they could be completed at the same time as the medical note. Furthermore, completion is likely to have been enhanced by the presence of a research assistant who reminded physicians of their task.

Based on this pilot trial, the planning of the recruitment of participants for a larger trial needs to be carefully thought out. Although demanding for principal investigators, having a meeting with members of the FMGs seems to be useful, as it could have reinforced the decision to participate in this type of study. Moreover, it could have led to a more informed consent, conducive to sustained participation. Second, recruiting patients particularly in the context of ARI also needs to be carefully planned. One of the data collection periods occurred during summertime, when patients consulting for ARIs are less frequent, and thus, the time for collection period was longer than anticipated. Third, the pilot study was limited to FMGs, as they had a fee-for-service organisation, but other primary care groups might be considered for a larger trial. Family practice teaching units could be an interesting group to target, as they are usually larger in size than FMGs and have more physicians, including residents. Regardless of study type or method, successful identification, selection, and recruitment of participants, whether they are groups of physicians, individual physicians, or patients, are important issues to be considered by all intending researchers [15], and pilot trials provide such opportunities.

Regarding participation in the DECISION+ program, a total of 46% of all participating family physicians completed the three 3-hour workshops, for a total of nine hours over four to six months. We had made *a priori *acceptability criteria that a 70% participation rate would be ideal for the success of a larger-scale study. Considerations need to be taken and lessons learned. Findings from a recent systematic review on the adoption of SDM by providers revealed that participation of providers in the intervention varied between 47% and 100%, with a median of 52%, independent of the length of the intervention [10]. Moreover, a recent study reported that a six-hour workshop for practitioners seems the ideal length of time, independent of numbers of workshops [42]. In retrospect, inserting three workshops of three hours over a four- to six-month period was probably too demanding on the part of family physicians in FMGs who usually have other hospital, educational, and administrative duties. In light of these results, having 69% of the family physicians participating in two or more workshops (six hours or more) in this pilot trial seems acceptable. The burden associated with the numbers and lengths of the workshops were also demanding on the trainers who could not comply with all competing demands, which affected the replicability of the intervention in the control group, as reported elsewhere [31].

Considering all these elements, the length and duration of the workshop should be considered when planning and conducting a larger trial to increase generalisability of the intervention. Introduction of a web-based or self-learning element, reduction of the length and/or numbers of workshops for both participants and trainers, and improvement of train-the-trainers strategies for collaborators needs to be considered.

Limitations need to be acknowledged. First, the pilot study was limited to FMGs, as they had a fee-for-service organisation, but other primary care groups might be considered for a larger trial. Second, the low response rate of FMGs may limit the generalisability of the findings, although the sociodemographic characteristics of participating family physicians were similar to the characteristics provided by the provincial registry (data not shown). Third, we lacked information regarding the number of patients that presented themselves at the outpatient clinics for an ARI and the total number of patients seen by physicians during the study period. Finally, it is possible that FMGs, family physicians, and patients who participated in the study might have had higher interest or motivation in the DECISION+ program than those who did not participate in the study.

Notwithstanding these limitations, rigorous pilot trials are meritorious projects, and their results should be subjected to the same level of scrutiny and requirements as those provided to the results of full-scale trials [28]. However, opinions vary regarding whether the results of the pilot trial should focus on the implementation processes, efficacy outcomes, or both [20]. Publication of findings of pilot trials could certainly help to inform other researchers about methodological or practical challenges related to such studies and does constitute a meaningful contribution to the literature [27,43,44].

## Conclusions

The present study has highlighted the significant practical and methodological issues in undertaking a clustered randomised trial involving family physicians and their patients while minimising burden on the normal state of the clinical encounters. This pilot trial will help to better define a larger cluster trial concerning the evaluation of an intervention targeting the improvement of the SDM process among family physicians and their patients regarding the use of antibiotics for treating ARIs in primary care.

## Competing interests

The authors declare that they have no competing interests.

## Authors' contributions

AL, who was a doctoral student at the time, participated in all stages of the study, was responsible for the analysis, and wrote the manuscript. FL and ML developed the research protocol, were responsible for the overall conduct of the study, and participated in the drafting of the manuscript. All authors contributed to the final version. AL is its guarantor. All authors read and approved the final draft.
